# Genome-Wide Association Study of 2,093 Cases With Idiopathic Polyneuropathy and 445,256 Controls Identifies First Susceptibility Loci

**DOI:** 10.3389/fneur.2021.789093

**Published:** 2021-12-17

**Authors:** Bendik S. Winsvold, Ioannis Kitsos, Laurent F. Thomas, Anne Heidi Skogholt, Maiken E. Gabrielsen, John-Anker Zwart, Kristian Bernhard Nilsen

**Affiliations:** ^1^Department of Research and Innovation, Division of Clinical Neuroscience, Oslo University Hospital, Oslo, Norway; ^2^K. G. Jebsen Center for Genetic Epidemiology, Department of Public Health and Nursing, Faculty of Medicine and Health Sciences, Norwegian University of Science and Technology, Trondheim, Norway; ^3^Department of Neurology, Oslo University Hospital, Oslo, Norway; ^4^Institute of Clinical Medicine, Faculty of Medicine, University of Oslo, Oslo, Norway; ^5^Department of Clinical and Molecular Medicine, Norwegian University of Science and Technology, Trondheim, Norway; ^6^BioCore–Bioinformatics Core Facility, Norwegian University of Science and Technology, Trondheim, Norway; ^7^Clinic of Laboratory Medicine, St. Olavs Hospital, Trondheim University Hospital, Trondheim, Norway

**Keywords:** polyneuropathies, genome-wide association study, genetics, population, peripheral nervous system diseases, genetic predisposition to disease

## Abstract

**Background:** About one third of patients with chronic polyneuropathy have no obvious underlying etiology and are classified as having idiopathic polyneuropathy. The lack of knowledge about pathomechanisms and predisposing factors limits the development of effective prevention and treatment for these patients. We report the first genome-wide association study (GWAS) of idiopathic polyneuropathy.

**Methods:** Cases with idiopathic polyneuropathy and healthy controls were identified by linkage to hospital records. We performed genome-wide association studies using genetic data from two large population-based health studies, the Trøndelag Health Study (HUNT, 1,147 cases and 62,204 controls) and UK Biobank (UKB, 946 cases and 383,052 controls). In a two-stage analysis design, we first treated HUNT as a discovery cohort and UK Biobank as a replication cohort. Secondly, we combined the two studies in a meta-analysis. Downstream analyses included genetic correlation to other traits and diseases. We specifically examined previously reported risk loci, and genes known to cause hereditary polyneuropathy.

**Results:** No replicable risk loci were identified in the discovery-replication stage, in line with the limited predicted power of this approach. When combined in a meta-analysis, two independent loci reached statistical significance (rs7294354 in *B4GALNT3, P*-value 4.51 × 10^−8^) and (rs147738081 near *NR5A2, P*-value 4.75 × 10^−8^). Idiopathic polyneuropathy genetically correlated with several anthropometric measures, most pronounced for height, and with several pain-related traits.

**Conclusions:** In this first GWAS of idiopathic polyneuropathy we identify two risk-loci that indicate possible pathogenetic mechanisms. Future collaborative efforts are needed to replicate and expand on these findings.

## Introduction

Polyneuropathy is a common and disabling condition resulting from the dysfunction of peripheral nerves. Typical symptoms include symmetrical distal numbness and paresthesia, often accompanied by pain and weakness (1). The diagnosis of polyneuropathy is established by history taking, clinical examination, and nerve conduction studies if available. Polyneuropathy has a prevalence of 1–3%, increasing to 7% in the elderly (1), and leads to considerable neurological impairment and a shortened life expectancy (2). Despite extensive testing, 12–49% of patients with polyneuropathy have no obvious underlying etiology (1, 3), making *idiopathic polyneuropathy* the most common neuropathy. The lack of knowledge about pathomechanisms and predisposing factors in this group of patients poses a considerable clinical challenge both in prevention and in the development of better treatments.

Genome-wide association studies (GWAS) offer a robust method for identifying genetic risk factors in complex disorders, and may be of particular interest in conditions where pathomechanisms are poorly understood. A challenge is that GWAS require genetic data from a large number of cases and controls, typically requiring the use of population studies or international consortium collaborations. Large population surveys seldom have the possibility to identify cases with polyneuropathy, as no available questionnaire can identify polyneuropathy with a high specificity. To our knowledge, no previous GWAS study has been performed for symptomatic idiopathic polyneuropathy. In this study we combined information from hospital records with self-reported measures and biological samples from two large population-based surveys; the Trøndelag health study (HUNT) and the UK Biobank. The aim of the study was to identify genetic variants associated with idiopathic polyneuropathy.

## Materials and Methods

### Patient Recruitment and Sample Collection

The Trøndelag Health Study (HUNT) consists of four different population-based health surveys conducted in the county of Nord-Trøndelag, Norway over ~35 years. For the present study we included participants from HUNT2 (1995–1997) and HUNT3 (2006–2008) (4). In each survey, the entire adult population (≥ 20 years) was invited to participate by completing questionnaires, attending clinical examinations and donate biological samples, including DNA. Participation rates in HUNT2 and HUNT3 were 69.5% (*n* = 65,237) and 54.1% (*n* = 50,807), respectively. The HUNT study has been described in detail elsewhere (4). The health care system in Norway is publicly funded. We obtained from hospital registries diagnosis codes according to the International Classification of Diseases, ninth and tenth revisions (ICD-9, ICD-10) for all inpatient and outpatient contacts between 1987 and 2018 for all genotyped participants in the HUNT study.

Details about the UK Biobank sample have been described elsewhere (5). Briefly, the cohort consists of 503,325 subjects enrolled between 2006 and 2010 throughout the United Kingdom. Age at baseline was between 40 and 59 years, and 94% were of self-reported European ancestry. At baseline, genome-wide genotyping was performed for samples from 488,377 individuals. Inpatient hospital data on all participants was available through electronic record linkage.

The study was conducted under UK biobank application no. 40135, and approved by the Regional Committee for Medical and Health Research Ethics, Norway (#2015/573/REK midt). Written informed consent was obtained from all participants.

### Idiopathic Polyneuropathy Phenotype

Identical criteria were used to define cases and controls in the HUNT and UK Biobank studies. Cases were defined by 1) the presence of at least one hospital contact with a registered diagnosis of idiopathic progressive neuropathy (ICD-10 G60.3, ICD-9 356.4), other specified idiopathic peripheral neuropathy (ICD-9 356.8), unspecified hereditary and idiopathic neuropathy (ICD-10 G60.9), or unspecified polyneuropathy (ICD-10 G62.9, ICD-9 356.9); 2) no hospital contact with a registered diagnosis of diabetes (ICD-10 E10–E14, ICD-9 250). Controls included all participants who had no hospital contacts with a registered diagnosis of hereditary or idiopathic polyneuropathy (ICD-10 G60, ICD-9 356), other inflammatory polyneuropathy (ICD-10 G61.8, ICD-9 357), unspecified inflammatory polyneuropathy (ICD-10 G61.9), other and unspecified polyneuropathies (ICD-10 G62), polyneuropathy in diseases classified elsewhere (ICD-10 G63), idiopathic peripheral autonomic neuropathy (ICD-10 G90.0), paraneoplastic neuropathy (ICD-10 G13.0), autonomic neuropathy in diseases classified elsewhere (ICD-10 G99.0), or diabetes (ICD-10 E10–E14, ICD-9 250).

The proportion of cases among the total sample was 1.81% in HUNT (1,147/63,351) and 0.25% in UK Biobank (946/383,998).

### Genotyping, Quality Control, and Imputation of the HUNT and UK Biobank Samples

A detailed description of genotyping, quality control and imputation of the HUNT sample is given in the Supplementary Material. Briefly, DNA from 71,860 HUNT samples was genotyped using the Illumina HumanCoreExome array. After rigorous quality control, data from 69,715 samples of recent European ancestry was imputed using a customized reference panel consisting of the Haplotype Reference consortium release 1.1 (HRC v1.1) and 2,201 low-coverage whole-genome sequences samples from HUNT. We excluded variants with imputation *r*^2^ < 0.3 or an estimated minor allele count (MAC) < 3 among cases, corresponding to minor allele count (MAF) < 0.0013. A total of 13,629,169 variants from 63,351 individuals (1,147 cases and 62,204 controls) were available for association analysis.

Genotyping, quality control and imputation of the UK Biobank sample have been described in detail elsewhere (5). We used the UK Biobank version 3 (March 2018) imputed genetic data. Briefly, samples were genotyped using the UK Biobank Axiom array and the UK BiLEVE Axiom array. The released genotype data has 805,426 markers. These were imputed with reference to the Haplotype Reference Consortium and UK10K haplotype resources. We excluded imputed variants with INFO score <0.3 or an estimated MAC < 3 among cases, corresponding to MAF < 0.0016. This resulted in 15,076,004 variants from 383,998 individuals (946 cases and 383,052 controls) being available for association analysis.

### Power Analysis and Overall Analysis Design

A discovery GWAS using only the larger HUNT study (1,147 cases and 62,204 controls) was predicted to give 48% power to detect associated variants with genotype relative risk (RR) ≥ 1.3, given an additive genetic model, disease prevalence 1.81% and disease allele frequency 0.3 (6). Applying the same statistical model, a combined analysis of HUNT and UK Biobank (2,093 cases and 445,256 controls) was predicted to give 97% power to detect associated variants with RR ≥ 1.3, and 79% power for RR ≥ 1.25. The true power will be somewhat lower if there is heterogeneity between the two studies (7). Given the limited power to detect associations of moderate effect size using one cohort alone, we decided from the outset on a two-stage design. First, we performed a discovery analysis in HUNT, using the UK Biobank sample for replication. For each independent significant locus (*P*-value < 5 × 10^−8^) in the discovery sample (HUNT) we selected the lead variant, or a proxy variant, for replication in the UK Biobank sample. Second, in order to maximize power for discovery of true risk variants we combined the two studies in a meta-analysis. All further downstream analyses were based on the results from the meta-analysis.

### Genome-Wide Association Analysis

Association analysis was performed using a mixed logistic regression model implemented in SAIGE (8) (version 0.35.8.3), where idiopathic polyneuropathy was modeled as the dependent variable, and the genetic variants as the independent variable. Dosages were used for imputed variants. For the HUNT analysis we included as covariates sex, birth year, analysis batch and the first four principal components. For the UK Biobank analysis we included as covariates sex, birth year, array and the first six principal components. For the X-chromosome analyses males were coded as diploid.

### Meta-Analysis of the HUNT and UK Biobank Association Studies

Prior to meta-analysis, summary statistics (including beta, standard error and *P*-value for each variant) from the two studies were harmonized using EasyQC with standard settings (9), including harmonization of alleles, exclusion of duplicate variants and comparison of allele frequencies between the datasets and with the HRC v1.1. Summary statistics were next meta-analyzed using METAL (10) with the “STDERR” option, weighing effect size estimates using the inverse of the corresponding standard errors. Only variants present in both studies were included, resulting in 11,530,364 variants in the final meta-analysis.

### Previously Reported Genetic Risk Loci for Polyneuropathy

We are not aware of previous genetic association studies of idiopathic polyneuropathy. From the GWAS catalog (11) we identified five previously reported genetic associations to other polyneuropathy phenotypes (12–16) (Supplementary Table 1). The reported index variants were tested for association to idiopathic polyneuropathy in our meta-analysis results. Association *P*-values were corrected for multiple comparisons by using the false discovery rate (FDR) method implemented in the R function p.adjust (17), correcting for five tests. FDR-corrected *P*-value < 0.05 was considered significant.

Secondly, we selected the 69,887 variants that resided within 20 kilobases from 175 genes related to monogenic forms of polyneuropathy (see the Supplemental Material), considering as significant an FDR-corrected *P*-value < 0.05, when correcting for 69,887 tests.

### Univariate LD-Score Regression

Linkage Disequilibrium (LD) Score Regression (LDSC v1.0.1) was used to estimate the proportion of a true polygenic signal vs. confounding factors such as population stratification, and to calculate single nucleotide polymorphism (SNP) based heritability (18). SNPs present in the HapMap 3 reference set were used, after excluding SNPs in the HLA region, and SNPs with large-effects, explaining >1% of phenotype variation, or variants in LD with such. Heritability estimates were converted to the liability scale assuming a population prevalence of idiopathic polyneuropathy of 1.81%, corresponding to the prevalence seen in the population-based HUNT study.

### Genetic Correlation

Using the same set of variants, genetic correlation analysis by LD Score regression (LDSC version 1.0.0) (19) was performed between idiopathic polyneuropathy and 774 diseases and traits for which GWAS results were available in LDHub (Supplementary Table 2) (20). Genetic correlation by LD score regression is not biased by sample overlap (19). The significance level for each correlation analysis was corrected for multiple testing by the FDR-method implemented in the R function p.adjust(), correcting for 774 tests (17). Traits correlating with idiopathic polyneuropathy with FDR < 0.05 were considered of interest, and are reported.

### Gene-Based and Tissue-Specificity Analysis

We performed gene-based association analysis as implemented in FUMA (21), with default settings. Gene mapping was based on MAGMA (v1.6) analysis (21). For MAGMA gene-set analysis, variants were assigned to the genes obtained from Ensembl build 85 (protein-coding genes). To test the relationship between the risk loci identified for idiopathic polyneuropathy and tissue-specific genetic expression, we examined their effect on the expression of genes up to one megabase away using FUMA expression quantitative trait locus (eQTL) mapping and tissue expression data from GTEx v8 (22). Lastly, we performed MAGMA tissue expression analysis and differentially expressed gene sets, based on the previous MAGMA gene-based *P*-values.

## Results

### Study Participants

In HUNT 1,147 participants qualified as cases (539 women, 618 men) and 62,204 as controls (33,368 women, 28,836 men). The mean age at recruitment in HUNT was 52.8 years (SD 17.5) overall, 62.9 years overall (SD 13.1) for cases and 52.6 years (SD 17.5) for controls. If the same individual participated in both HUNT2 and HUNT3, the age at HUNT3 is reported. In UK Biobank 946 participants qualified as cases (416 women, 530 men) and 383,052 as controls (210,904 women, 172,148 men). The mean age at recruitment in UK Biobank was 56.7 years (SD 8.0) overall, 60.0 years (SD 7.0) for cases and 56.7 years (SD 8.0) for controls.

### Discovery and Replication Analysis

Using HUNT as the discovery sample, three variants at three independent loci showed genome-wide significant (*P*-value < 5 × 10^−8^) associations with idiopathic polyneuropathy (Supplementary Table 3; Supplementary Figures 1, 2), all being rare (MAF < 0.5%) imputed variants having large association effect sizes (OR > 10), and sparse surrounding LD-structure (Supplementary Figures 1B–D). For two of the variants (rs1425573669 and rs188798445) their validity could not be determined as neither they, nor any proxies in high LD, were present in the replication sample. The third variant (rs11784454 in *PSD3*) was present in the replication sample, but did not show association with idiopathic polyneuropathy (*P*-value 0.65).

The genome-wide results are summarized for HUNT in Supplementary Figures 1, 2, and for UK Biobank in Supplementary Figures 3, 4. The genomic inflation factor (λ) was 1.024 for the analysis in HUNT and 1.008 for the analysis in UK Biobank.

### Meta-Analysis

**Figure 1:**
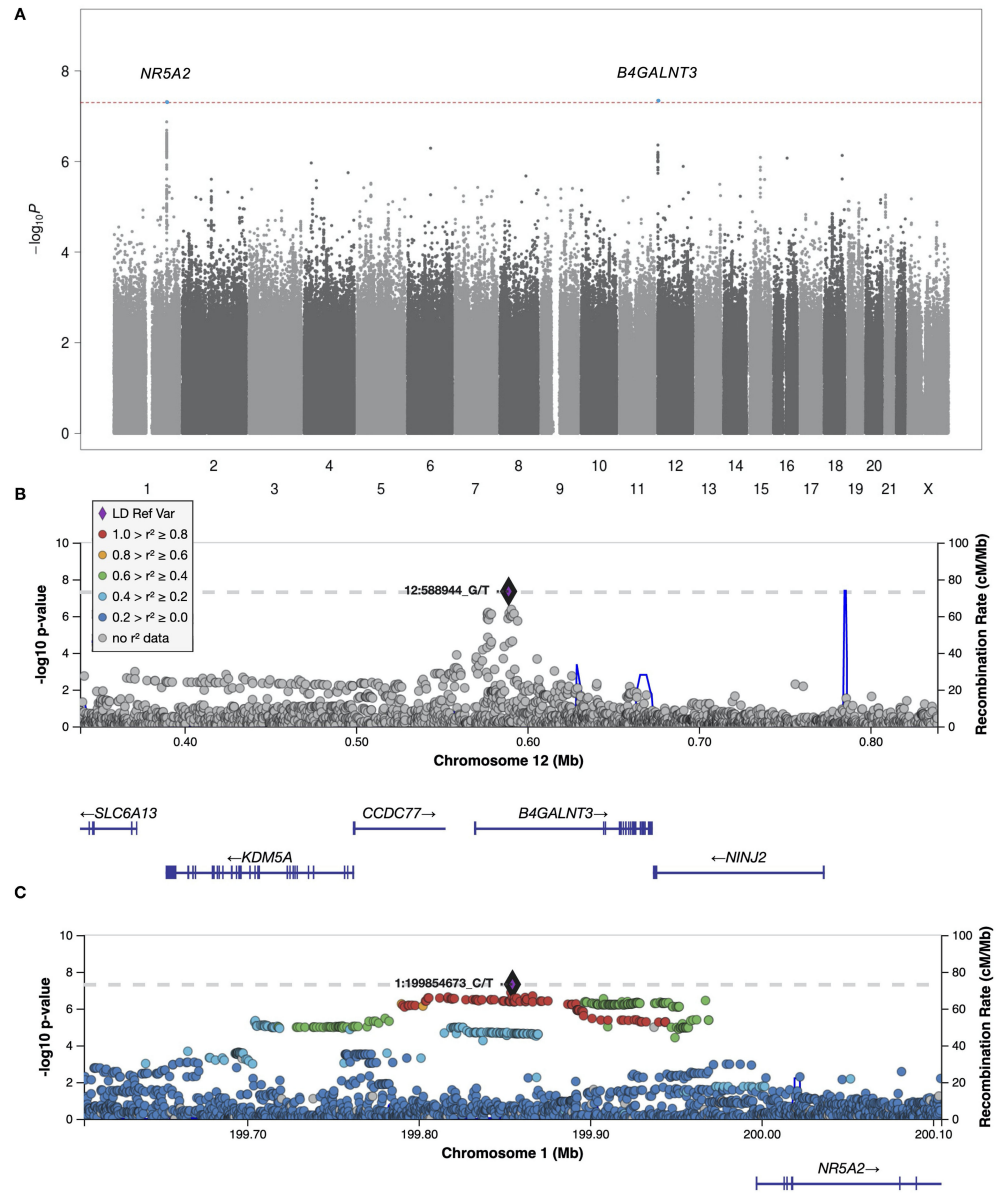
Manhattan plot and regional plots for the meta-analysis. **(A)** Manhattan plot showing results of the meta-analysis of all idiopathic polyneuropathy samples (2,093 cases and 383,998 controls). Each dot represents a genetic variant. The horizontal axis gives the genomic coordinate and the vertical axis the significance level (−log_10_ *p*-value). Markers that reached genome-wide significance (*P* < 5 × 10^−8^) are highlighted in blue, and annotated with the nearest protein coding gene. **(B,C)** Regional Manhattan plots of the two genome-wide significant idiopathic neuropathy loci. The index variant for each locus is marked with a purple diamond and annotated with its corresponding location number (CRCh37/hg19). Variants are colored based on their linkage disequilibrium (*r*^2^) with the labeled lead variant in 1000 Genomes (EUR) data, according to the legend (of note, the index variant at locus #1 is not present in the 1000 Genomes dataset). The solid blue line shows the local recombination rate. Gencode genes are shown. Figures were obtained from LocusZoom (25). Positions are given as in build GRCh37/hg19.

**Figure 2:**
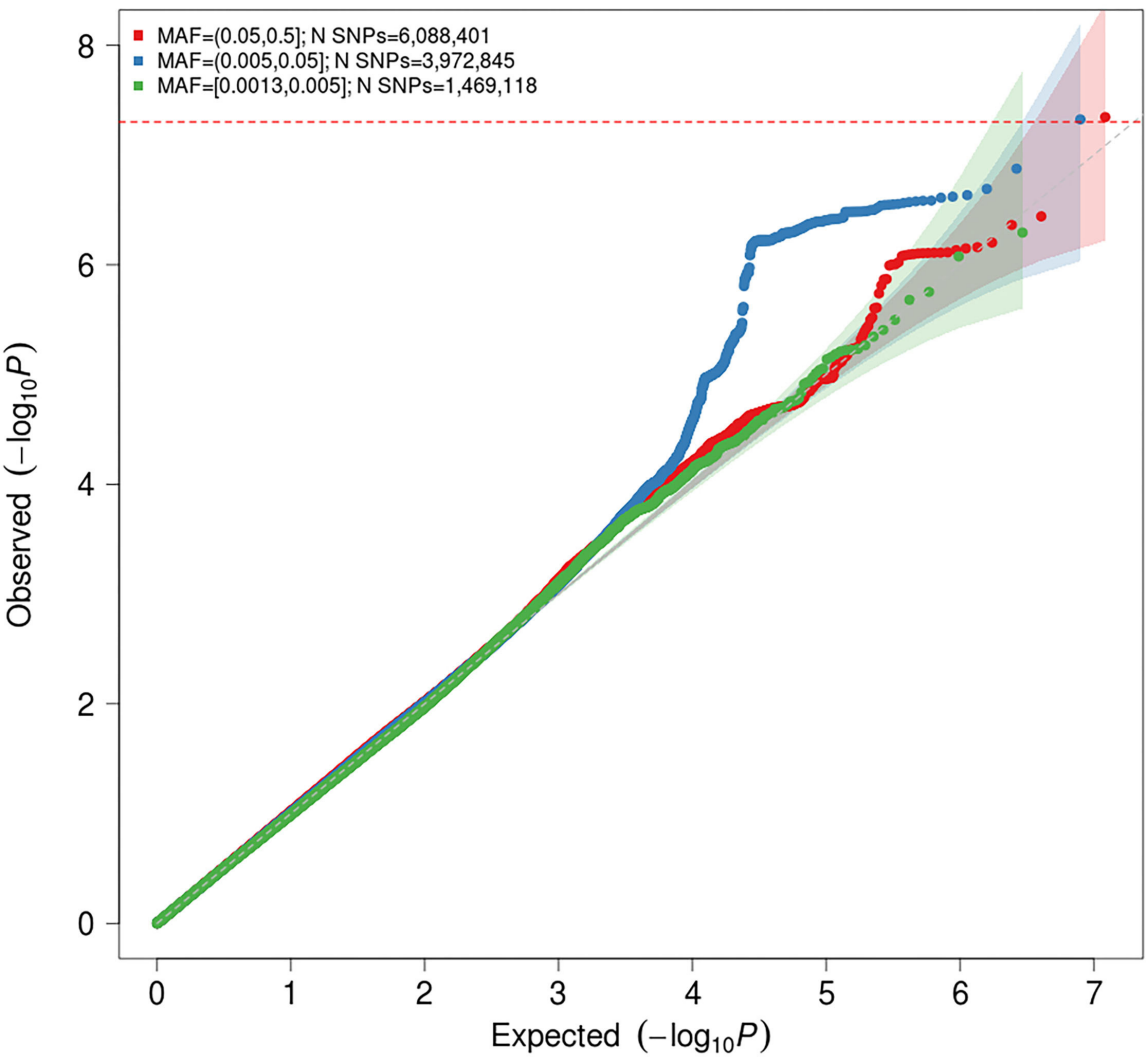
Quantile-quantile (Q-Q) plot for association with idiopathic polyneuropathy in the meta-analysis. The horizontal axis shows –log10 *P*-values expected under the null distribution. The vertical axis shows observed –log10 *P*-values. The shaded areas represents the 95% confidence intervals of expected *P*-values under the null hypothesis. Red = common variants (minor allele frequency [MAF] ≥ 0.05); blue = low frequency variants (MAF = 0.005–0.05); green = rare variants (MAF < 0.005). Genomic inflation factor (λ) = 1.020.

We next meta-analyzed the HUNT and UK Biobank samples, including only variants that were present in both studies, resulting in 11,530,364 tested variants from 2,093 cases and 445,256 controls. Two variants at two independent loci reached genome-wide significance, hereafter named locus #1 (chromosome 12, index variant rs7294354, *P*-value 4.51 × 10^−8^) and locus #2 (chromosome 1, index variant rs147738081, *P*-value 4.75 × 10^−8^) as sorted by *P*-value (Figures 1, 2; Table 1). The index variants at the two loci have MAF of 42.8% and 3.1%, respectively, and represent broader association loci involving several variants with a clear LD structure (Figures 1B,C). Locus #1 covers several genes, with the index variant being intragenic in *B4GALNT3* (Figure 1B). At locus #2 the index variant is located 142 kb upstream of the gene *NR5A2* (Figure 1C). The combined explained variance of the two significant variants was 0.71%.

**Table 1:** Details of the two loci associated with idiopathic neuropathy in the meta-analysis.

	**Locus #1**	**Locus #2**
Chromosome	12	1
Position	588944	199854673
rsID	rs7294354	rs147738081
Nearest gene(s)^†^	*B4GALNT3*	*NR5A2; PTPRC*
eQTL mapped genes	*B4GALNT3; RAD52*	*INAVA*
Effect (risk) allele	T	T
Other allele	G	C
		**Meta-analysis**
EAF	0.428	0.031
OR (95% CI)	1.19 (1.12–1.27)	1.68 (1.39–2.02)
*P*	4.51 × 10^−8^	4.75 × 10^−8^
		**HUNT**
EAF	0.423	0.024
EAF cases	0.462	0.038
EAF controls	0.423	0.024
OR (95% CI)	1.18 (1.08–1.28)	1.83 (1.39–2.43)
*P*	1.50 × 10^−4^	2.13 × 10^−5^
		**UK Biobank**
EAF	0.434	0.037
EAF cases	0.479	0.052
EAF controls	0.434	0.037
OR (95% CI)	1.20 (1.10–1.32)	1.57 (1.22–2.01)
*P*	7.51 × 10^−5^	4.14 × 10^−4^

*OR (odds ratio), 95% CI (confidence interval) and P (P-value) for the association between the genetic variant and idiopathic polyneuropathy. Position, genome position (build hg19/GRCh37); rsID, dbSNP Reference; eQTL, expression quantitative trait locus; EAF, effect allele frequency*.

† *For variants not located in a gene, the two nearest genes are listed, separated by semicolon*.

The genomic inflation factor (λ) for the meta-analysis was 1.023, while the LD score regression intercept was 1.0028 (SE 0.006), with a ratio of 0.11 (SE 0.24), indicating that 89% of the observed signal is caused by true polygenic heritability rather than by confounding factors, such as population stratification. The estimated SNP-based heritability (*h*^2^) of idiopathic polyneuropathy was 8.9% (SE 3.36%) on the liability scale. It should be noted that the modest sample sizes do not allow for robust estimation of SNP-based heritability, and this estimate should be interpreted with caution.

Fine mapping with PICS identified the index variant (rs7294354) at locus #1 to be the most likely causal variant, with a causal probability of 0.34. No other single variants at either of the two loci had a causal PICS-probability of >0.2.

### Previously Reported Polyneuropathy Loci

None of the five loci previously associated with polyneuropathy phenotypes showed association to idiopathic polyneuropathy in our study (Supplementary Table 1). Also, none of the 69,887 variants near 175 genes related to monogenic forms of polyneuropathy showed significant association after correcting for multiple testing.

### Genetic Correlation of Idiopathic Polyneuropathy With Somatic and Psychiatric Traits

Among the 774 diseases and traits tested, idiopathic polyneuropathy was genetically correlated with 31 traits with FDR < 0.05 (Table 2; Supplementary Table 2). Among these 31 traits, 19 (61%) were anthropometric measures, with idiopathic polyneuropathy being positively correlated with larger body size. Among the anthropometric measures the highest correlation was seen for height (genetic correlation coefficient, r_g_ 0.30). Six of the traits (19%) were pain related, with idiopathic polyneuropathy being positively correlated with pain.

**Table 2:** Genetic correlations of idiopathic polyneuropathy with diseases and traits on LDHub.

**Trait**	**Class**	**Source**	**r_**g**_ (SE)**	***P***	**P_**FDR**_**
Basal metabolic rate	Metabolism	UKB	0.27 (0.08)	3.0 × 10^−4^	0.028
Weight	Anthropometric	UKB	0.25 (0.07)	4.0 × 10^−4^	0.028
Leg fat-free mass (right)	Anthropometric	UKB	0.27 (0.08)	4.0 × 10^−4^	0.028
Leg predicted mass (right)	Anthropometric	UKB	0.27 (0.08)	4.0 × 10^−4^	0.028
Leg fat-free mass (left)	Anthropometric	UKB	0.27 (0.08)	4.0 × 10^−4^	0.028
Leg predicted mass (left)	Anthropometric	UKB	0.27 (0.08)	4.0 × 10^−4^	0.028
Whole body fat-free mass	Anthropometric	UKB	0.26 (0.08)	5.0 × 10^−4^	0.028
Height	Anthropometric	PMID 20881960	0.30 (0.09)	6.0 × 10^−4^	0.028
Comparative height size at age 10	Anthropometric	UKB	0.26 (0.08)	6.0 × 10^−4^	0.028
Overall health rating	Overall health	UKB	0.31 (0.09)	6.0 × 10^−4^	0.028
Leg pain on walking	Pain	UKB	0.54 (0.16)	6.0 × 10^−4^	0.028
Whole body water mass	Anthropometric	UKB	0.26 (0.08)	6.0 × 10^−4^	0.028
Arm predicted mass (left)	Anthropometric	UKB	0.26 (0.08)	6.0 × 10^−4^	0.028
Standing height	Anthropometric	UKB	0.24 (0.07)	7.0 × 10^−4^	0.028
Long-standing illness, disability or infirmity	Overall health	UKB	0.35 (0.10)	7.0 × 10^−4^	0.028
Arm fat-free mass (left)	Anthropometric	UKB	0.25 (0.07)	7.0 × 10^−4^	0.028
Trunk predicted mass	Anthropometric	UKB	0.26 (0.08)	7.0 × 10^−4^	0.028
ICD-10 diagnosis R07 Pain in throat and chest	Pain	UKB	0.55 (0.16)	7.0 × 10^−4^	0.028
Taking other prescription medications	Overall health	UKB	0.37 (0.11)	8.0 × 10^−4^	0.028
Arm fat-free mass (right)	Anthropometric	UKB	0.26 (0.08)	8.0 × 10^−4^	0.028
Arm predicted mass (right)	Anthropometric	UKB	0.26 (0.08)	8.0 × 10^−4^	0.028
Trunk fat-free mass	Anthropometric	UKB	0.26 (0.08)	8.0 × 10^−4^	0.028
Hip circumference	Anthropometric	UKB	0.23 (0.07)	9.0 × 10^−4^	0.029
Trunk fat mass	Anthropometric	UKB	0.22 (0.07)	9.0 × 10^−4^	0.029
Medication for pain relief, constipation, heartburn: Ibuprofen (e.g., Nurofen)	Pain	UKB	0.50 (0.15)	1.0 × 10^−3^	0.031
Pain type(s) experienced in last month: None of the above	Pain	UKB	−0.37 (0.11)	1.0 × 10^−3^	0.033
Frequency of tenseness/restlessness in last 2 weeks	Other	UKB	0.39 (0.12)	1.4 × 10^−3^	0.040
Frequency of tiredness/lethargy in last 2 weeks	Other	UKB	0.31 (0.10)	1.5 × 10^−3^	0.041
Chest pain or discomfort	Pain	UKB	0.36 (0.12)	1.9 × 10^−3^	0.047
Whole body fat mass	Anthropometric	UKB	0.20 (0.06)	1.9 × 10^−3^	0.047
Medication for pain relief, constipation, heartburn: None of the above	Pain	UKB	−0.33 (0.11)	1.9 × 10^−3^	0.047

*Traits correlated with idiopathic polyneuropathy with a false discovery rate (FDR) < 0.05 are shown, grouped in “classes” defined post-hoc to aid interpretation. Source, source of GWAS summary statistics for the given trait, that are used in the correlation analysis; UKB, UK Biobank; PMID, Pubmed ID for publication; rg, genetic correlation coefficient; SE, standard error; P, P-value; P_FDR_, FDR-corrected P-value*.

### Downstream Bioinformatics Analysis

Using FUMA gene-based eQTL annotation mapping based on data from GTEx v8 (21, 22), we identified two genes mapping to locus #1, *B4GALNT3* (eQTL in amygdala of the brain) and *RAD52* (eQTL in tibial nerve, aorta, coronary and tibial artery, left ventricle of heart, ovary, salivary gland, adipose visceral omentum, basal ganglia of brain, esophageal mucosa and thyroid), and one gene, *INAVA*, mapping to locus #2 (eQTL in leukocytes) (Table 1).

Variant associations were also mapped to 18,912 protein coding genes using the gene-based test implemented in FUMA. No gene was significantly associated with idiopathic polyneuropathy after FDR-correction for multiple testing. The strongest associations were seen for *B4GALNT3* (locus #1, *P*-value 6.36 × 10^−6^, p_FDR_ 0.085), *CNFN* (chromosome 19, *P*-value 8.96 × 10^−6^, p_FDR_ 0.085) and *ATP5S* (chromosome 14, *P*-value 1.66 × 10^−5^, p_FDR_ 0.10). Finally, MAGMA (21) gene set and tissue expression analyses did not show significant enrichment of association signals to any specific gene set or tissue (Supplementary Figure 5; Supplementary Table 4).

## Discussion

We report the first GWAS of idiopathic polyneuropathy, based on data from two large population-based studies linked to hospital records. We applied a two-stage analysis design. First, treating HUNT as a discovery cohort and UK Biobank as a replication cohort, no replicable risk variants were identified, in line with the limited predicted power of this approach. Second, when combining the two studies in a meta-analysis of 2,093 cases and 445,256 controls, we identified two independent genome-wide significant risk loci. Each locus involved multiple associated variants showing a clear LD structure, and LD score regression suggested that the overall association signal, while modest, was largely (89%) due to true polygenic heritability rather than to confounding factors such as population stratification. The SNP-based heritability of idiopathic polyneuropathy was estimated at 8.9%. There was moderate genetic correlation of idiopathic polyneuropathy with large body size, most pronounced for body height, and with several pain-related traits.

Locus #1 covers several genes (Figure 1B). While bioinformatics analyses cannot pinpoint the causal gene, one of these genes is of particular interest. *NINJ2* encodes Ninjurin2 (Nerve Injury-Induced Protein 2). In the peripheral nervous system, Ninjurin2 is upregulated in Schwann cells surrounding the distal segment of injured nerves, promoting neurite outgrowth, and is believed to have a role in nerve regeneration after nerve injury (23). Circulating levels of Ninjurin2 have been associated with nerve injury, particularly with nerve recovery (24). It has also been found that *in vitro* downregulation of *NINJ2* induces apoptosis and leads to reduced survival of neurons, while upregulation protects neurons from hydrogen peroxide-induced cell death and apoptosis (25). These findings suggest that *NINJ2* acts as a pro-survival factor in human neurons. Interestingly, *NINJ2* was recently identified in a genome-wide DNA methylation study, where diabetic autonomic neuropathy was associated with increased methylation, and a corresponding decreased expression, of *NINJ2* (26). The involvement of *NINJ2* in idiopathic polyneuropathy is highly plausible. A suggested mechanism would be that reduced function or expression of Ninjurin2 leads to impaired nerve regeneration, promoting the development of polyneuropathy.

Among the other genes at locus #1, *KDM5A* (Lysine Demethylase 5A) should also be mentioned. The encoded protein is an epigenetic regulator which has been implicated in neurodevelopmental disorders, including autism (27–29). A recent study in *Drosophila* larvae indicated that *KDM5* is a key transcription regulator of genes important for synaptic structure and function (27), and a *KDM5A* knockout mouse exhibited abnormal dendritic morphogenesis of cortical neurons and cognitive deficits (28). The role of *KDM5A* in the peripheral nervous system remains to be elucidated.

Locus #2 is located in a gene-poor region. The nearest gene, *NR5A2*, encodes liver receptor homolog 1 (LRH-1), which is involved in various biological processes, including bile acid metabolism, glucose homeostasis (30), and carcinogenesis (31). Its potential mechanism in polyneuropathy is unclear. However, the locus is also an eQTL (i.e., it is predicted to affect expression levels) in leukocytes for the gene *INAVA*, which encodes Innate Immunity Activator Protein. Genetic variants in *INAVA* have been associated with inflammatory bowel disease, and it seems to have a critical role in regulating macrophage function (32). Macrophages have a central role in autoimmune polyneuropathy (33), and some evidence suggests they are implicated in age-related and diabetic polyneuropathy (34, 35). While the possible implication of *INAVA* is interesting, follow-up studies will be needed to determine if it has a causal role in idiopathic polyneuropathy.

We find it interesting that large body size, in particular height, showed genetic correlation with idiopathic polyneuropathy (rg 0.30). This supports some studies that have reported height to be an important predictor for symptomatic polyneuropathy, the assumed link being increased axon surface exposure to toxins (36). It should be noted that height is also known to affect nerve conduction velocity (37, 38), which is used to diagnose polyneuropathy. While we consider it less likely, an alternative explanation of the observed genetic correlation could be that laboratory reference values were not adequately adjusted for height.

Idiopathic polyneuropathy also genetically correlated with several pain-related traits, including leg pain on walking, throat and chest discomfort, and the overall use of pain medications. Pain is a prominent feature in a proportion of patients with chronic polyneuropathy. While the prevalence of neuropathic pain among patients with idiopathic polyneuropathy has not been reported, it is 30–60% among patients with idiopathic axonal polyneuropathy (39, 40), and 40–50% in patients with diabetic polyneuropathy (41). Our results may indicate shared biological mechanisms between symptomatic idiopathic polyneuropathy and other pain-related traits. One possibility is that mechanisms involved in a more general susceptibility for chronic pain also predispose to the development of painful polyneuropathy, which in turn increases the likelihood of receiving a hospital diagnosis. The observed correlations could also result if idiopathic polyneuropathy is a substantial cause for pain among cases in each of the pain-trait GWAS studies we used for comparison.

When specifically examining variants in or near 175 genes from a genetic panel of known hereditary polyneuropathies we did not find any robust associations, making it unlikely that these genes have a major role in the risk for idiopathic polyneuropathy. Also, we did not replicate any of the five loci reported to be associated with other types of polyneuropathy in previous GWAS studies, including drug induced and diabetic polyneuropathy (12–16). This may indicate that these other types of polyneuropathy have a different genetic predisposition than idiopathic polyneuropathy.

A strength of this study was the large number of participants and the unbiased approach, using hospital-based diagnoses and healthy controls from large population-based health studies. This increases the external validity of the findings. A limitation is that the clinical phenotypes probably are diverse. Structured clinical information and the exact basis for diagnosis could not be obtained, which reduces the internal validity of the findings. While in HUNT the prevalence of idiopathic polyneuropathy (1.8%) was in line with previous population estimates, the observed prevalence in UK Biobank was substantially lower than expected (0.25%). A major reason for this discrepancy is likely that only inpatient diagnoses were used in UK Biobank, and most patients with polyneuropathy will not need inpatient treatment. The difference in age distribution between the two cohorts is unlikely to explain the discrepancy, since the mean age at participation was slightly lower in HUNT than in UK Biobank, and the prevalence of polyneuropathy tends to increase with age. Currently no available questionnaire can identify polyneuropathy with a high specificity. This limitation can be overcome by future studies where a more detailed neurological phenotyping of the subjects is performed.

In order to reach sufficient power, this will likely require international collaboration. In the current study, sample sizes were insufficient to obtain internal replication of the findings, and the reported associations will need to be replicated in independent cohorts. Larger studies will also be needed for robust downstream analyses, which can give a more complete picture of the biological processes involved in idiopathic polyneuropathy. The results from the present study gives the first genetic clues to the biological basis for idiopathic polyneuropathy and should motivate larger genetic studies that include more fine-grained clinical phenotyping.

In conclusion, in this first GWAS of idiopathic polyneuropathy we identify two associated loci, suggesting possible pathomechanisms including impaired nerve regeneration and inflammatory pathways, and find genetic correlation with several anthropometric measures and pain-related traits. Future studies are needed to replicate and expand on these findings.

## Data Availability Statement

The HUNT dataset presented in this article is not readily available because of privacy restrictions (the Regional Committee for Medical and Health Research Ethics, Norway #2015/573/REK midt). The data is however available on request for projects with an ethical approval. Requests to access the dataset should be directed to HUNT Research Centre, Forskningsveien 2, 7600 Levanger, Norway; Phone +47 74 07 51 80; email kontakt@hunt.ntnu.no The UK Biobank data analyzed in this study (version 3, March 2018) was obtained from the UK Biobank (application no. 40135). This dataset is freely available to researchers by application to the UK Biobank through their web-based application system: https://www.ukbiobank.ac.uk/enable-your-research/apply-for-access.

## Ethics Statement

The studies involving human participants were reviewed and approved by REK SørØst. The patients/participants provided their written informed consent to participate in this study.

## The Hunt All-in Pain Group

Amy E Martinsen^1, 2, 3^, Ben M Brumpton^3^, Cristen J Willer^4^, Egil Andreas Fors^5^, Elisabeth Gjefsen^1^, Espen Saxhaug Kristoffersen^6, 7, 1^, Ingrid Heuch^1^, Ingunn Mundal^8^, Jonas Bille Nielsen^3, 4, 9^, Kjersti Storheim^10, 11^, Knut Hagen^12^, Kristian Hveem^3, 13, 14^, Lars G. Fritsche^15^, Linda M Pedersen^1^, Marie Udnesseter Lie^2, 1^, Oddgeir L Holmen^13^, Sigrid Børte^2, 3, 1^, Synne Øien Stensland^10, 16^, Wei Zhou^17, 18^

^1^Department of Research and Innovation, Division of Clinical Neuroscience, Oslo University Hospital, Oslo, Norway. ^2^Institute of Clinical Medicine, Faculty of Medicine, University of Oslo, Oslo, Norway. ^3^K. G. Jebsen Center for Genetic Epidemiology, Department of Public Health and Nursing, Faculty of Medicine and Health Sciences, Norwegian University of Science and Technology (NTNU), Trondheim, Norway. ^4^Department of Internal Medicine, Division of Cardiovascular Medicine, University of Michigan, Ann Arbor, MI, 48109, USA. ^5^Department of Public Health and Nursing, Faculty of Medicine and Health Sciences, Norwegian University of Science and Technology (NTNU), Trondheim, Norway. ^6^Department of General Practice, University of Oslo, Oslo, Norway. ^7^Department of Neurology, Akershus University Hospital, Lørenskog, Norway. ^8^Department of Health Science, Molde University College, Molde, Norway. ^9^Department of Epidemiology Research, Statens Serum Institut, Copenhagen, Denmark. ^10^Research and Communication Unit for Musculoskeletal Health (FORMI), Department of Research and Innovation, Division of Clinical Neuroscience, Oslo University Hospital, Oslo, Norway. ^11^Department of physiotherapy, Faculty of Health Sciences, Oslo Metropolitan University, Oslo, Norway. ^12^Department of Neuromedicine and Movement Science, Faculty of Medicine and Health Sciences, Norwegian University of Science and Technology (NTNU), Trondheim, Norway. ^13^HUNT Research Center, Department of Public Health and Nursing, Faculty of Medicine and Health Sciences, Norwegian University of Science and Technology (NTNU), Trondheim, Norway. ^14^Department of Research, Innovation and Education, St. Olavs Hospital, Trondheim University Hospital, Trondheim, Norway. ^15^Center for Statistical Genetics, Department of Biostatistics, University of Michigan, Ann Arbor, MI, 48109, USA. ^16^Norwegian Centre for Violence and Traumatic Stress Studies, Oslo, Norway. ^17^Department of Computational Medicine and Bioinformatics, University of Michigan, Ann Arbor, MI, 48109, USA. ^18^Analytic and Translational Genetics Unit, Massachusetts General Hospital, Boston, MA, USA.

## Author Contributions

BW, IK, KN, and J-AZ designed the study and interpretation of data. BW, IK, LT, AS, and MG performed analyses. BW and IK drafted the manuscript. All authors revised the work and approve of the final version.

## Conflict of Interest

The authors declare that the research was conducted in the absence of any commercial or financial relationships that could be construed as a potential conflict of interest.

## Publisher's Note

All claims expressed in this article are solely those of the authors and do not necessarily represent those of their affiliated organizations, or those of the publisher, the editors and the reviewers. Any product that may be evaluated in this article, or claim that may be made by its manufacturer, is not guaranteed or endorsed by the publisher.
