# Supplementary material for: Genome-Wide Association Study of 2,093 Cases With Idiopathic Polyneuropathy and 445,256 Controls Identifies First Susceptibility Loci
Source: Front Neurol. 2021 Dec 17;12:789093. doi: 10.3389/fneur.2021.789093 (PMC8718917; doi:10.3389/fneur.2021.789093)
Supplement: Supplementary file 2 [file Table_2.DOCX]

Supplementary Material

[1 Supplementary Methods 1](#_Toc86395743)

[1.1 Genotyping, quality control and imputation of the HUNT sample 1](#_Toc86395744)

[1.2 Analysis of variants near genes related to monogenic forms of polyneuropathy 2](#_Toc86395745)

[2 Supplementary Tables 4](#_Toc86395746)

[Supplementary Table 1. Previous genetic associations to polyneuropathy 4](#_Toc86395747)

[Supplementary Table 2. Genetic correlations of idiopathic polyneuropathy with diseases and traits on LDHub (full results) 5](#_Toc86395748)

[Supplementary Table 3. Summary of the three genomic loci associated with idiopathic polyneuropathy in the discovery sample 6](#_Toc86395749)

[Supplementary Table 4. MAGMA gene set enrichment analysis 7](#_Toc86395750)

[3 Supplementary Figures 8](#_Toc86395751)

[Supplementary Figure 1. Manhattan plot and regional plots for the genome-wide association analysis of idiopathic polyneuropathy in HUNT (1,147 cases and 62,204 controls). 8](#_Toc86395752)

[Supplementary Figure 2. Quantile-quantile (Q-Q) plot for the genome-wide association analysis of idiopathic polyneuropathy in HUNT (1,147 cases and 62,204 controls). 10](#_Toc86395753)

[Supplementary Figure 3. Manhattan plot for the genome-wide association analysis of idiopathic polyneuropathy in UK Biobank (946 cases and 383,052 controls). 11](#_Toc86395754)

[Supplementary Figure 4. Quantile-quantile (Q-Q) plot for the genome-wide association analysis of idiopathic polyneuropathy in UK Biobank (946 cases and 383,052 controls). 12](#_Toc86395755)

[Supplementary Figure 5. MAGMA Tissue Expression Analysis 13](#_Toc86395756)

[4 References 13](#_Toc86395757)

# Supplementary Methods

## 1.1 Genotyping, quality control and imputation of the HUNT sample

DNA from 71,860 HUNT samples was genotyped using one of three different Illumina HumanCoreExome arrays (HumanCoreExome12 v1.0, HumanCoreExome12 v1.1 and UM HUNT Biobank v1.0). Samples that failed to reach a 99% call rate, had contamination > 2.5% as estimated with BAF Regress (1), large chromosomal copy number variants, lower call rate of a technical duplicate pair and twins, gonosomal constellations other than XX and XY, or whose inferred sex contradicted the reported gender, were excluded. Samples that passed quality control were analyzed in a second round of genotype calling following the Genome Studio quality control protocol described elsewhere (2). Genomic position, strand orientation and the reference allele of genotyped variants were determined by aligning their probe sequences against the human genome (Genome Reference Consortium Human genome build 37 and revised Cambridge Reference Sequence of the human mitochondrial DNA; http://genome.ucsc.edu) using BLAT (3). Variants were excluded if their probe sequences could not be perfectly mapped, cluster separation was < 0.3, Gentrain score < 0.15, showed deviations from Hardy Weinberg equilibrium in unrelated samples of European ancestry with P-value < 0.0001), had a call rate < 99%, or another assay with higher call rate genotyped the same variant. Ancestry of all samples was inferred by projecting all genotyped samples into the space of the principal components of the Human Genome Diversity Project (HGDP) reference panel (938 unrelated individuals; downloaded from http://csg.sph.umich.edu/chaolong/LASER/) (4, 5), using PLINK. Recent European ancestry was defined as samples that fell into an ellipsoid spanning exclusively European populations of the HGDP panel. The different arrays were harmonized by reducing to a set of overlapping variants and excluding variants that showed frequency differences > 15% between data sets, or that were monomorphic in one and had MAF > 1% in another data set. The resulting genotype data were phased using Eagle2 v2.3 47 (6).

Imputation was performed on the 69,715 samples of recent European ancestry using Minimac3 (v2.0.1,<http://genome.sph.umich.edu/wiki/Minimac3>) (7) with default settings (2.5 Mb reference based chunking with 500kb windows) and a customized Haplotype Reference consortium release 1.1 (HRC v1.1) for autosomal variants and HRC v1.1 for chromosome X variants (8). The customized reference panel represented the merged panel of two reciprocally imputed reference panels: (1) 2,201 low-coverage whole-genome sequences samples from the HUNT study and (2) HRC v1.1 with 1,023 HUNT WGS samples removed before merging. We excluded imputed variants with Rsq < 0.3 or an estimated minor allele count < 3 among cases, corresponding to minor allele count (MAF) < 0.0013.

A total of 13,629,169 variants from 63,351 individuals (1,147 cases and 62,204 controls) were available for association analysis.

## 1.2 Analysis of variants near genes related to monogenic forms of polyneuropathy

We specifically examined variants within 20 kilobases of 175 genes with known relation to monogenic forms of polyneuropathy. These genes correspond to the 175 genes that are present on the gene panel for hereditary polyneuropathy currently used at our University hospital (https://genetikkportalen.no/?act=genpan&katID=19&GpanID=7). The panel was generated based on three separate gene panels developed by Genomics England (<https://panelapp.genomicsengland.co.uk/>): 1) Familial dysautonomia (V 1.8) (https://panelapp.genomicsengland.co.uk/panels/7/), 2) Hereditary neuropathy (V 1.368:) (https://panelapp.genomicsengland.co.uk/panels/85/), and 3) Pain syndromes (V 1.9): (https://panelapp.genomicsengland.co.uk/panels/288/). The 175 genes are *AAAS*, *AARS1*, *ABCA1*, *ABHD12*, *AGTPBP1*, *AGXT*, *AIFM1*, *AP1S1*, *APOA1*, *APTX*, *ARHGEF10*, *ARSA*, *ATL1*, *ATL3*, *ATM*, *ATP1A1*, *ATP7A*, *B4GALNT1*, *BAG3*, *BCKDHB*, *BICD2*, *BSCL2*, *C12orf65*, *CD59*, *CHCHD10*, *CNTNAP1*, *COA7*, *COX6A1*, *CPOX*, *CTDP1*, *CYP27A1*, *DARS2*, *DCTN1*, *DEGS1*, *DNAJB2*, *DNAJC3*, *DNM2*, *DNMT1*, *DRP2*, *DST*, *DYNC1H1*, *EGR2*, *ELP1*, *ERCC6*, *ERCC8*, *ETFDH*, *FAH*, *FAM126A*, *FBLN5*, *FBXO38*, *FGD4*, *FIG4*, *FLVCR1*, *FXN*, *GALC*, *GAN*, *GARS1*, *GBA2*, *GDAP1*, *GJB1*, *GJC2*, *GLA*, *GMPPA*, *GNB4*, *HADHA*, *HADHB*, *HARS1*, *HINT1*, *HK1*, *HMBS*, *HSPB1*, *HSPB8*, *IARS2*, *IGHMBP2*, *INF2*, *KCNA2*, *KIF1A*, *KIF5A*, *LITAF*, *LMNA*, *LRSAM1*, *LYST*, *MCM3AP*, *MFN2*, *MMACHC*, *MME*, *MORC2*, *MPV17*, *MPZ*, *MTMR2*, *MTTP*, *MYH14*, *NAGA*, *NAGLU*, *NDRG1*, *NEFH*, *NEFL*, *NGF*, *NTRK1*, *OPA1*, *OPA3*, *PDHA1*, *PDYN*, *PEX10*, *PEX7*, *PHOX2B*, *PHYH*, *PLEKHG5*, *PLP1*, *PMM2*, *PMP2*, *PMP22*, *PNKP*, *PNPLA6*, *POLG*, *POLR3A*, *PPOX*, *PRDM12*, *PRKCG*, *PRNP*, *PRPS1*, *PRX*, *PTEN*, *PTPN11*, *PTRH2*, *RAB7A*, *REEP1*, *RETREG1*, *SACS*, *SBF1*, *SBF2*, *SCARB2*, *SCN10A*, *SCN11A*, *SCN9A*, *SCYL1*, *SEPTIN9*, *SETX*, *SH3TC2*, *SIGMAR1*, *SLC12A6*, *SLC25A19*, *SLC25A46*, *SLC52A2*, *SLC52A3*, *SLC5A7*, *SMN1*, *SOX10*, *SPAST*, *SPG11*, *SPG7*, *SPTLC1*, *SPTLC2*, *SUCLA2*, *SURF1*, *SYT2*, *TFG*, *TRIM2*, *TRPA1*, *TRPV4*, *TTPA*, *TTR*, *TUBB3*, *TWNK*, *TYMP*, *VCP*, *VPS13A*, *VRK1*, *WARS1*, *WNK1*, *XK*, *XPA*, *XRCC1*, *YARS1*, *ZFYVE26*.

# Supplementary Tables

## Supplementary Table 1. Previous genetic associations to polyneuropathy

| **Previously reported PN loci** | | | | | | | **Association with PN in the current study** | |
| --- | --- | --- | --- | --- | --- | --- | --- | --- |
| **Reported trait** | **Nearest gene** | **rsID** | **EA** | **OR (95%CI)** | **P** | **Ref** | **OR (95%CI)** | **P**† |
| Sural nerve amplitude | *PRPH* | rs73112142 | A | 0.77  (0.67-0.88) | 1.14E-11 | (9) | 1.10 (0.79-1.52) | 0.59 |
| Response to anti-retroviral therapy (ddI/d4T) in HIV-1 infection (Grade 3 peripheral neuropathy) | *IL2RA* | rs12722486 | Not reported | 38.2  (6.6-219.6) | 1.50E-09 | (10) | 1.01 (0.87-1.18) | 0.89 |
| Vincristine-related peripheral neuropathy in children with acute lymphoblastic leukemia | *CEP72* | rs924607 | T | 2.43  (1.70-3.49) | 6.33E-09 | (11) | 1.02 (0.96-1.09) | 0.49 |
| Docetaxel-induced peripheral neuropathy in metastatic castrate-resistant prostate cancer | *VAC14* | rs875858 | Not reported | 3.60  (2.21-5.84) | 2.12E-08 | (12) | 1.00 (0.86-1.16) | 1.00 |
| Diabetic peripheral neuropathy in type 2 diabetes | *XIRP2* | rs13417783 | T | 0.63  (0.55-0.72) | 7.90E-12 | (13) | 1.00 (0.91-1.08) | 0.91 |

Previous GWAS catalog associations with polyneuropathy (index variants) tested in results from GWAS meta-analysis of idiopathic polyneuropathy using HUNT and UK Biobank samples. OR (odd ratio) with 95% confidence interval (CI) and P-value (P) in the original report and in the current study are shown. rsID = dbSNP Reference. EA = effect allele. †Uncorrected P-value is shown. For all five variants, the FDR-corrected P-value was 1.00.

## Supplementary Table 2. Genetic correlations of idiopathic polyneuropathy with diseases and traits on LDHub (full results)

[ Uploaded separately as a Microsoft Excel spreadsheet ]

## Supplementary Table 3. Summary of the three genomic loci associated with idiopathic polyneuropathy in the discovery sample

| **Chromosome** | 8 | 8 | 9 |
| --- | --- | --- | --- |
| **Position** | 17697266 | 18424272 | 15825803 |
| **rsID** | rs1425573669 | rs11784454 | rs188798445 |
| **Nearest gene(s)†** | *MTUS1; FGL1* | *PSD3* | *CCDC171* |
| **Effect (risk) allele** | G | A | G |
| **Other allele** | C | T | C |
| **Discovery sample (HUNT)** | | | |
| **Imputation R^2^** | 0.86 | 0.97 | 0.83 |
| **EAF** | 0.0016 | 0.0038 | 0.0022 |
| **OR** | 53.52 | 10.59 | 30.88 |
| **95% CI** | 13.57 - 211.03 | 4.74 - 23.66 | 9.16 - 104.08 |
| **P** | 1.15 x10^-8^ | 1.04 x10^-8^ | 2.60 x10^-8^ |
| **Replication sample (UKB)** | | | |
| **Imputation R^2^** | Na | 0.97 | Na |
| **EAF** | Na | 0.0021 | Na |
| **OR** | Na | 0.79 | Na |
| **95% CI** | Na | 0.29-2.20 | Na |
| **P** | Na | 0.65 | Na |

OR (odds ratio), 95% CI (confidence interval) and P (P-value) for the association between the genetic variant and idiopathic polyneuropathy. Imputation R^2^ = imputation quality metric (R^2^) (all the variants were imputed). Chr = chromosome. Pos = genome position (build hg19/GRCh37). rsID = dbSNP Reference. EAF = effect allele frequency. HUNT = The Trøndelag Health Study. UKB = UK Biobank. Na = not available. †For variants not located in a gene, the two nearest genes are listed, separated by semicolon.

## Supplementary Table 4. MAGMA gene set enrichment analysis

| **Gene Set** | **N genes** | β | **SE_β_** | **SE** | **P** | **Pbon** |
| --- | --- | --- | --- | --- | --- | --- |
| GO_mf:go_damaged_dna_binding | 67 | 0.392 | 0.023 | 0.093 | 1.18 x 10^-5^ | 0.18 |
| Curated_gene_sets:pid_hdac_classii_pathway | 34 | 0.584 | 0.025 | 0.150 | 4.72 x 10^-5^ | 0.73 |
| GO_cc:go_catenin_complex | 29 | 0.721 | 0.028 | 0.185 | 4.97 x 10^-5^ | 0.77 |
| GO_bp:go_cellular_response_to_glucose_starvation | 41 | 0.490 | 0.023 | 0.126 | 5.19 x 10^-5^ | 0.80 |
| GO_bp:go_tube_lumen_cavitation | 5 | 1.568 | 0.025 | 0.427 | 1.22 x 10^-4^ | 1 |
| GO_mf:go_1_phosphatidylinositol_binding | 15 | 0.866 | 0.024 | 0.238 | 1.40 x 10^-4^ | 1 |
| Curated_gene_sets:reactome_recycling_of_bile_acids_and_salts | 16 | 0.788 | 0.023 | 0.219 | 1.57 x 10^-4^ | 1 |
| Curated_gene_sets:reactome_noncanonical_activation_of_notch3 | 8 | 0.870 | 0.018 | 0.244 | 1.85 x 10^-4^ | 1 |
| GO_bp:go_negative_regulation_of_myoblast_differentiation | 24 | 0.605 | 0.022 | 0.172 | 2.24 x 10^-4^ | 1 |
| GO_bp:go_aromatic_amino_acid_transport | 8 | 1.003 | 0.021 | 0.288 | 2.52 x 10^-4^ | 1 |

β, SE (standard error), P (P-value) and Pbon (Bonferroni corrected P-value) for the MAGMA gene set enrichment analysis is shown. The top 10 gene sets are displayed. The gene sets used by MAGMA were obtained from MsigDB v5.2. In total 10894 gene sets (Curated gene sets: 4728, GO terms:6166) were tested. Curated gene sets consist of 9 data resources including KEGG, Reactome and BioCarta (http://software.broadinstitute.org/gsea/msigdb/collection_details.jsp#C2 for details). GO terms consists of three categories, biological processes (bp), cellular components (cc) and molecular functions (mf). All parameters were set as default (competitive test).

# Supplementary Figures

s****

## Supplementary Figure 1. Manhattan plot and regional plots for the genome-wide association analysis of idiopathic polyneuropathy in HUNT (1,147 cases and 62,204 controls).

(A) Each dot represents a genetic variant. The horizontal axis gives the genomic coordinate and the vertical axis the significance level (-log_10_ *p* value). Markers that reached genome-wide significance (*P* < 5 × 10^−8^) are highlighted in blue, and annotated with the nearest protein coding gene. (B-D) Regional Manhattan plots of the three genome-wide significant idiopathic neuropathy loci. The index variant for each locus is marked with a purple diamond and annotated with its corresponding location number (CRCh37/hg19). Variants are colored based on their correlation (*r^2^*) with the labelled lead variant in 1000 Genomes (EUR) data, according to the legend. The solid blue line shows the local recombination rate. Gencode genes are shown. Figures were obtained from LocusZoom (14). Positions are given as in build GRCh37/hg19.

*
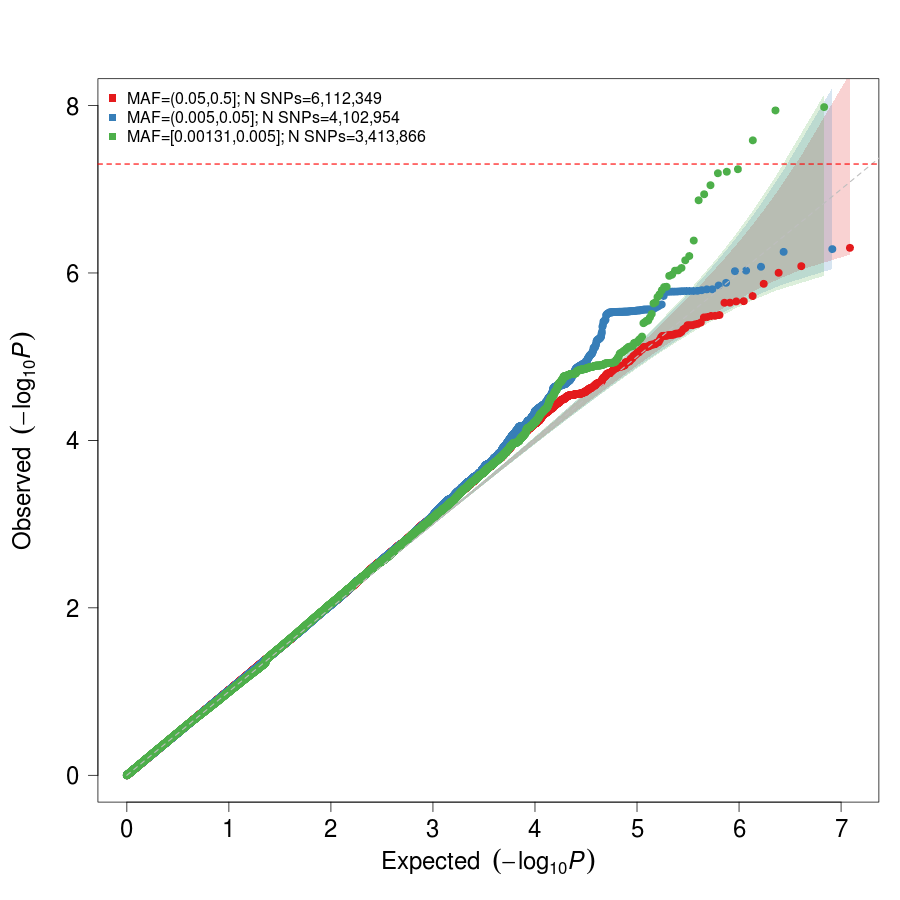
*

## Supplementary Figure 2. Quantile-quantile (Q-Q) plot for the genome-wide association analysis of idiopathic polyneuropathy in HUNT (1,147 cases and 62,204 controls).

The horizontal axis shows -log10 P-values expected under the null distribution. The vertical axis shows observed -log10 P-values. The shaded areas represent the 95% confidence intervals of expected P-values under the null hypothesis. Red = common variants (minor allele frequency [MAF] ≥ 0.05); blue = low frequency variants (MAF = 0.005–0.05); green = rare variants (MAF < 0.005). Genomic inflation factor (λ) = 1.024.

## Supplementary Figure 3. Manhattan plot for the genome-wide association analysis of idiopathic polyneuropathy in UK Biobank (946 cases and 383,052 controls).

Each dot represents a genetic variant. The horizontal axis gives the genomic coordinate and the vertical axis the significance level (-log_10_ *p* value). No marker reached genome-wide significance (*P* < 5 × 10^−8^).


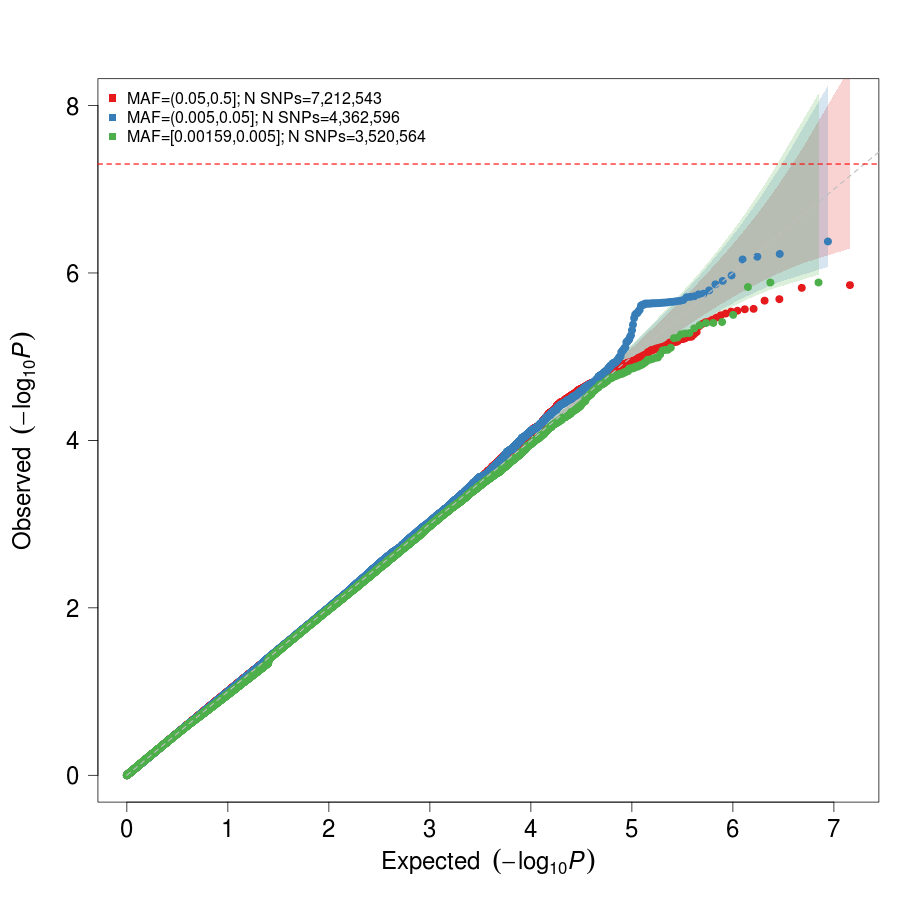


## Supplementary Figure 4. Quantile-quantile (Q-Q) plot for the genome-wide association analysis of idiopathic polyneuropathy in UK Biobank (946 cases and 383,052 controls).

The horizontal axis shows -log10 P-values expected under the null distribution. The vertical axis shows observed -log10 P-values. The shaded areas represent the 95% confidence intervals of expected P-values under the null hypothesis. Red = common variants (minor allele frequency [MAF] ≥ 0.05); blue = low frequency variants (MAF = 0.005–0.05); green = rare variants (MAF < 0.005). Genomic inflation factor (λ) = 1.008.

##
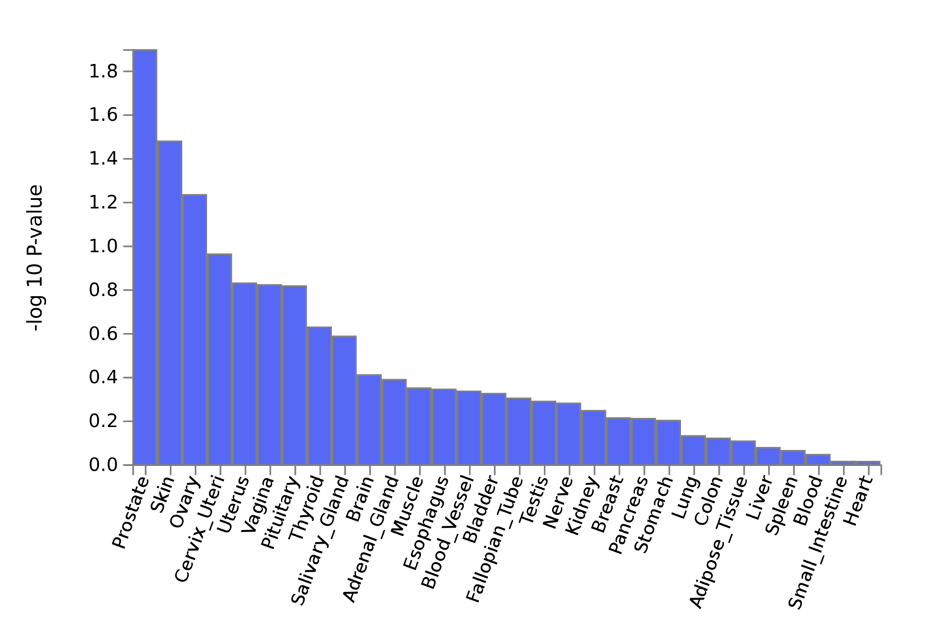
Supplementary Figure 5. MAGMA Tissue Expression Analysis

The MAGMA tissue expression analysis first calculates gene-based P-values based on variants located within genes from Ensembl build 85), amd mext performs a one-sided test for a positive relationship between these gene-based P-values and tissue-specific gene expression in tissue types in the GTEx v8 RNA-seq data (for details https://fuma.ctglab.nl/tutorial#magma).

# References

1. Jun G, Flickinger M, Hetrick KN et al. Detecting and estimating contamination of human DNA samples in sequencing and array-based genotype data. Am J Hum Genet. 2012;91:839-848.

2. Guo Y, He J, Zhao S et al. Illumina human exome genotyping array clustering and quality control. Nat Protoc. 2014;9:2643-2662.

3. Encode Project Consortium. An integrated encyclopedia of DNA elements in the human genome. Nature. 2012;489:57-74.

4. Li JZ, Absher DM, Tang H et al. Worldwide human relationships inferred from genome-wide patterns of variation. Science. 2008;319:1100-1104.

5. Wang C, Zhan X, Bragg-Gresham J et al. Ancestry estimation and control of population stratification for sequence-based association studies. Nat Genet. 2014;46:409-415.

6. Loh PR, Danecek P, Palamara PF et al. Reference-based phasing using the Haplotype Reference Consortium panel. Nat Genet. 2016;48:1443-1448.

7. Das S, Forer L, Schonherr S et al. Next-generation genotype imputation service and methods. Nat Genet. 2016;48:1284-1287.

8. McCarthy S, Das S, Kretzschmar W et al. A reference panel of 64,976 haplotypes for genotype imputation. Nat Genet. 2016;48:1279-1283.

9. Bjornsdottir G, Ivarsdottir EV, Bjarnadottir K et al. A PRPH splice-donor variant associates with reduced sural nerve amplitude and risk of peripheral neuropathy. Nat Commun. 2019;10:1777.

10. Leger PD, Johnson DH, Robbins GK et al. Genome-wide association study of peripheral neuropathy with D-drug-containing regimens in AIDS Clinical Trials Group protocol 384. J Neurovirol. 2014;20:304-308.

11. Diouf B, Crews KR, Lew G et al. Association of an inherited genetic variant with vincristine-related peripheral neuropathy in children with acute lymphoblastic leukemia. JAMA. 2015;313:815-823.

12. Hertz DL, Owzar K, Lessans S et al. Pharmacogenetic Discovery in CALGB (Alliance) 90401 and Mechanistic Validation of a VAC14 Polymorphism that Increases Risk of Docetaxel-Induced Neuropathy. Clin Cancer Res. 2016;22:4890-4900.

13. Tang Y, Lenzini PA, Pop-Busui R et al. A Genetic Locus on Chromosome 2q24 Predicting Peripheral Neuropathy Risk in Type 2 Diabetes: Results From the ACCORD and BARI 2D Studies. Diabetes. 2019;68:1649-1662.

14. Pruim RJ, Welch RP, Sanna S et al. LocusZoom: regional visualization of genome-wide association scan results. Bioinformatics. 2010;26:2336-2337.
